# PCaAnalyser: A 2D-Image Analysis Based Module for Effective Determination of Prostate Cancer Progression in 3D Culture

**DOI:** 10.1371/journal.pone.0079865

**Published:** 2013-11-20

**Authors:** Md Tamjidul Hoque, Louisa C. E. Windus, Carrie J. Lovitt, Vicky M. Avery

**Affiliations:** 1 Computer Science, University of New Orleans, Louisiana, United States of America; 2 Discovery Biology, Eskitis Institute for Drug Discovery, Griffith University, Brisbane, Queensland, Australia; CSIR-Institute of Microbial Technology, India

## Abstract

Three-dimensional (3D) *in vitro* cell based assays for Prostate Cancer (PCa) research are rapidly becoming the preferred alternative to that of conventional 2D monolayer cultures. 3D assays more precisely mimic the microenvironment found *in vivo*, and thus are ideally suited to evaluate compounds and their suitability for progression in the drug discovery pipeline. To achieve the desired high throughput needed for most screening programs, automated quantification of 3D cultures is required. Towards this end, this paper reports on the development of a prototype analysis module for an automated high-content-analysis (HCA) system, which allows for accurate and fast investigation of *in vitro* 3D cell culture models for PCa. The Java based program, which we have named PCaAnalyser, uses novel algorithms that allow accurate and rapid quantitation of protein expression in 3D cell culture. As currently configured, the PCaAnalyser can quantify a range of biological parameters including: nuclei-count, nuclei-spheroid membership prediction, various function based classification of peripheral and non-peripheral areas to measure expression of biomarkers and protein constituents known to be associated with PCa progression, as well as defining segregate cellular-objects effectively for a range of signal-to-noise ratios. In addition, PCaAnalyser architecture is highly flexible, operating as a single independent analysis, as well as in batch mode; essential for High-Throughput-Screening (HTS). Utilising the PCaAnalyser, accurate and rapid analysis in an automated high throughput manner is provided, and reproducible analysis of the distribution and intensity of well-established markers associated with PCa progression in a range of metastatic PCa cell-lines (DU145 and PC3) in a 3D model demonstrated.

## Introduction

Prostate cancer (PCa) has the highest prevalence of cancer in Australia, with nearly 20,000 new cases diagnosed each year [1]. At the onset of PCa, treatment involves androgen ablation, which temporarily slows progression, however recurrence of the cancer in an androgen-independent form is common [2]. At this stage, PCa can no longer be controlled by standard therapies, metastasis occurs, which is the major cause of mortality. Hence, new therapies are required to combat the disease prior to metastatic progression.

The importance of using 3D models in the evaluation of tumour development has previously been described [3], [4]. We, and others, have shown that 3D cultures afford a better platform for the study of solid tumour masses as tumour cells in this microenvironment discern antigenic profiles and phenotypic behaviour that mimic more precisely tumour cells as found *in vivo* [3], [4]. 3D cell culture allows for the subtle interplay of cells of the same or different origins within a matrix, mimicking cell-cell and cell-matrix interactions similar to those found *in vivo*. Moreover, proper alignment and spatial organisation in 3D is essential for tumour progression [5]. Taken together, these results suggest that 3D cultures may serve as a more biologically relevant model in the drug discovery pipeline.

Antigenic profiles of tumours excised from advanced PCa patients have identified alterations in the expression of numerous proteins. Of these, the androgen receptor (AR) [6], α6 [7], [8] and β1 integrin subunits [9], and more recently chemokine receptor CXCR4 [10] expression have been linked to increased Gleason grade and metastatic dissemination in PCa. Patient tumours consistently show an up-regulation of the β1 integrin subunit [11] and the chemokine receptor CXCR4 [12], accompanied by a redistribution and down regulation of α6 integrin [7], [8].

Heavily implicated in PCa bone metastases development and progression is the integrin β1 subunit [13]–[15]. Expression of α5β1 and α2β1 on PCa cells has been reported to facilitate interactions with bone stromal cells [15] and to actively promote invasion and adherence of PCa cells to the bone stroma *in vitro* [14] and experimental bone metastases *in vivo* [13]. Similarly the laminin-binding integrin α6β1 has been shown to permit extravasation of human PCa cells from circulation to the bone stroma *in vivo* [16]–[18].

Similarly, studies have indicated that the chemokine, CXCL12, plays a role in trafficking PCa cells to the bone. CXCL12 is expressed by stromal cells in target organs of PCa metastasis (bone, brain, lymph), but not in other tissues [19] and its receptor, CXCR4, is highly expressed by bone metastatic PCa cells [20], [21]. It was the aim of the current study to evaluate and analyse the expression patterns and distribution of these well-established markers associated with PCa progression, utilising a 3D model in conjunction with high throughput imaging analysis.

Another highly influential protein that contributes to the development of PCa is the AR [6]. The AR belongs to a superfamily of nuclear receptors and mediates the action of androgens such as 5-α-dihydrotestosterone (DHT). The AR and its activating ligands play an important role in PCa progression by mediating the responses of androgens and activating gene transcription. Although many of the well characterised effects of AR in PCa cells are reliant on the genomic effects which involve the transcription of target genes, non-genomic effects of androgens also influence cell behaviour. These include the activation of kinase cascades and cytoskeletal rearrangement which can stimulate cell motility [22]–[24].

Previously, we have reported that PCa PC3 metastatic cells re-express non-transcriptionally active AR which is in part mediated by the Src pathway [4]. Utilising a 3D model in conjunction with high throughput imaging analysis, it was a further aim of the current paper to evaluate the potential functional relevance of endogenous AR up-regulation in this cell line and how it may affect other important protein constituents known to mediate PCa progression including β1 integrin.

The ability to accurately analyse multiple imaging parameters obtained from 3D cell culture is to date reliant on highly specialised programs that are by no means automated. The existing imaging software suffers largely from the inherent problem of an inability to rapidly adapt and accommodate changing requirements effectively [25]. Here, we have developed an automated image-analysis based software named “PCaAnalyser” that is capable of analysing a range of parameters measured in 3D cell culture based on 2D images.

PCaAnalyser has been developed as an ImageJ [26]–[28] plugin, therefore has the capability to share and enhance several basic functions provided in ImageJ. The analysis undertaken by PCaAnalyser is a composition of two major algorithmic-interfaces. In the first step, the boundary of the cellular 3D spheroid is detected and the required masks are generated. In the second step, nuclei are detected and spheroid-memberships are then predicted using the masks and the boundaries. Similar approaches are followed to detect and study cytoplasmic areas by segregating them from critical noise.

The paradigm of PCaAnalyser, including the reporting component, has been designed to be flexible to enable the user to readily manipulate related analysis in a variety of ways, in addition to the default options.

With respect to the efficiency of PCaAnalyser, we have incorporated a candidate-membership based algorithm to speed-up the nucleus-spheroid detection process, making the overall processing time considerably faster. Time complexity analysis has been provided in this article, to assist with estimations of the processing time, which is based on the available data-parameters, such as number of spheroids per image, number of nuclei per spheroid and perimeter of the nucleus. This feature also provides a basis for comparison of the PCaAnalyser with other published algorithms.

In the current study, we utilised a Perkin Elmer Opera™ [29], a high throughput confocal imaging system, to generate the output from a PCa 3D cell culture model in microtitre plate format suitable for HTS. Complete reconstruction of the spheroids in 3D was memory and time intensive, thus 2D-image acquisition of the 3D objects, along the *xy*-plane, was applied as an alternative. In this population of spheroids, the 2D-image of the 3D objects varied in image resolution and sharpness due to the different focal planes, thus physical depths, as well as composition of the different cellular components of the 3D objects, which collectively made segmentation and detection challenging. Detection of various co-localised and multiple-contextual objects within the same channel-image also posed significant challenges. PCaAnalyser has been designed to successfully address such challenges. Thus, the PCaAnalyser presented here provides a valuable resource for investigations using 3D cell based models, particularly for use in high throughput automated systems.

Utilising PCaAnalyser, we report here the successful analysis of the distribution and intensity of well-established markers associated with PCa progression in a range of metastatic PCa cell-lines (DU145 and PC3) in a 3D model. Specifically, we have shown that in response to the ligand, SDF-1α, CXCR4 distribution and expression changed, indicative of a functional receptor. Moreover, we present here novel data concerning the down-regulation of β1 integrin after treatment with DHT. These results suggest that in PC3 cells, non-transcriptionally active AR can mediate other important proteins associated with PCa progression. These results have far reaching implications regarding AR targeted therapeutics in late-stage PCa treatment.

## Materials and Methods

### 1. Cancer Cell Lines

The DU145, PC3 and MDA-MB-231 cell lines were purchased from *American Tissue Culture Centre* (ATCC). The PCa Du145 and PC3 cell-lines, were maintained in RPMI-1640 (Invitrogen), supplemented with 10% fetal bovine serum (FBS, Gibco). The Breast Cancer (BCa) cell-line MDA-MB-231 was maintained in DMEM-F12 (Invitrogen), supplemented with 10% FBS. All cells were propagated at 37°C in standard cell culture conditions (5% CO_2_, 37°C) in T75 Flasks. Media was replenished every 3 days. Once cells had reached 80–90% confluency they were replated (1/10) in T75 flasks. After 10–12 passages, cells were discontinued.

### 2. Miniaturised 3D Cell Cultures

For the PCa cell lines, cells were plated on top of a 3D matrix gel bed (Matrigel: BD Bioscience) in glass-bottomed 96 well plates (Matrical: PerkinElmer). For miniaturised 3D cultures, wells were filled with 60 µl Matrigel™/culture medium (70%) and polymerised at 37°C with 5% CO_2_ for 1 hr. Cells were then seeded at ∼5000 cells per well and maintained as previously described above. Media was carefully removed and replenished every three days. Cultures were maintained for up to 12 days. For the BCa cell line MDA-MB-231, 1000 cells per well were plated on top of 15 µl of Growth Factor Reduced Matrigel (GFR Matrigel) in a 384-well CellCarrier plate (PerkinElmer).

### 3. Ligand and Drug Treatment Assays

Using a 96-well plate format PC3 cells were grown in 3D Matrigel cultures as described above. After 9 days in culture, 3D cells were treated with a natural androgen Dihydrotestosterone (DHT, Sigma-Aldrich) for 30 hrs in serum free media at 0, 1, 5 and 10 nM concentrations. Alternatively, 3D cultures were serum starved for 16 hrs and then treated with a CXCR4 ligand: SDF-1α: (30 ng/ml, R&D Systems) for 0, 20 and 40 mins. Cells were then fixed and processed for immunocytochemistry.

In the case of MDA-MB-231, cells were incubated for 3 days before application of 720 nM of Doxorubicin (Sigma-Aldrich) for 72 hrs. To view the nucleus in these cells Hoechst (1∶500, Invitrogen) was applied for 2 hrs before live cell imaging was undertaken. Doxorubicin emits endogenous fluorescence (excitation wavelength 480 nm, emission wavelength 530 nm).

### 4. Immunohistochemistry

The image based assay was undertaken as described previously [4] with minor modifications. Briefly, after 10 days in culture, 3D cultures of PCa cells DU145 were washed with PBS and fixed with PFA (4%, 10 minutes for 2D, 20 minutes for 3D), washed twice with PBS and blocked for 2 hrs with 2% BSA, 0.1% Triton-X, 0.05% TWEEN. Primary mouse anti-α6 and anti-β1 integrin subunit antibodies (5 µg/mL, R&D Systems) or mouse anti-CXCR4 (5 µg/mL, R&D Systems) were then added for 24 hours at 4°C in blocking buffer. Cells were washed with PBS (3×5 mins) and incubated at room temperature (RT) for 4 hrs with secondary antibodies (5 µg/mL 488 goat anti-mouse) and Hoechst nuclear stain (1/1000, Invitrogen).

### 5. Acquisition of Image

All fixed cells were imaged using the PerkinElmer Opera™ Quadruple Excitation High Sensitivity Confocal Cell Imager with a PerkinElmer 20/.75 water iris. Images were acquired using the 488 and 405 emission spectrum. Live cell imaging was completed using the PerkinElmer Opera using the 10× air objective with excitation by the 405 and 561 nm lasers. The acquired images were used as the input for the PCaAnalyser software for the analytical study described herein.

## Results

### 1. Channel Information and Challenges

In this instance, images via two fluorescent channels were investigated: (1) Ch-1, to detect the nucleus (Hoerchst: emission 405) and (2) Ch-2, to detect the expression of the protein of interest, (CXCR4, αb or β1 integrin subunits) and distribution.

Ch-1 is used to identify (a) the nucleus, and (b) the area of the spheroid. For extracting information pertaining to either the nucleus and/or the area of the spheroid the images from this channel were treated as bright-field images, which enable two different contexts of the image to be extracted from the same signal.

The image content has its own complexity as well: even though a confocal imaging system is employed, the spheroids have a 3D structure which incorporates depth and variation, resulting in uneven illumination of the focal plane. 3D spheroids are grown in a semi-solid gel, and as such they sit in a multitude of different z-planes. Thus, there are a relatively small number of cells that are imaged in focus within that focal plane. These images are comprised of a combination of both well-defined and ill-defined structures and blurred ill-defined components, thus providing a considerable challenge to accurately detect the nucleus of each cell. This becomes even more problematic when using automated analysis, as these signals are often integrated into the final output or intensity. Thus, greater control over threshold levels and the ability to filter parameters within the software was required to obtain accurate representative data.

Ch-2 provides the images which define the cell membrane and the cytoplasm of the individual cells of the spheroid mass. Images acquired through this channel have a low signal-to-noise ratio (SNR). The challenges with these specific images are (a) to segment, identify and read the zero or low intensity area along with the higher intensity area of the cytoplasm, (b) to develop and define suitable functions to classify various regions of cytoplasm, and (c) to avoid noise. The staining of any given immunofluorescence tag has with it a range of SNR values. Nuclear stains (Ch-1 images) are generally measured within the spectrum range of the 405 nm wavelength, which in comparison to the 488 nm (green) or 594 nm (red) spectrums, are highly permeable stains. Thus, Ch-1 images have less noise in comparison to those obtained with Ch-2. In addition, the Opera™ system is an automated high throughput confocal imager, whereby variable parameters could not be set for individual images, thus a particular setting sometimes works better for 6 to 10 images but not for the remainder. Therefore, our software was customized to address these problems, as well as reducing unwanted noise.

### 2. Analysis of Image

Our PCaAnalyser was developed as a plug-in of ImageJ [26]–[28], which provides an excellent environment for customisation, as well as easy access to many different image-file-formats due to LOCI plug-in and the Bio-Formats Java library [30], [31]. A compressed version of the associated FLEX files has been generated, which is the format primary image files are obtained in. FLEX file is the default file-format generated by the PerkinElmer Opera™ system that we have used to capture the raw images. These are compatible with MBF_ImageJ [32] (version of ImageJ) through extended supports of LOCI Bio-Formats (http://loci.wisc.edu/bio-formats/imagej).

**Figure 1:**
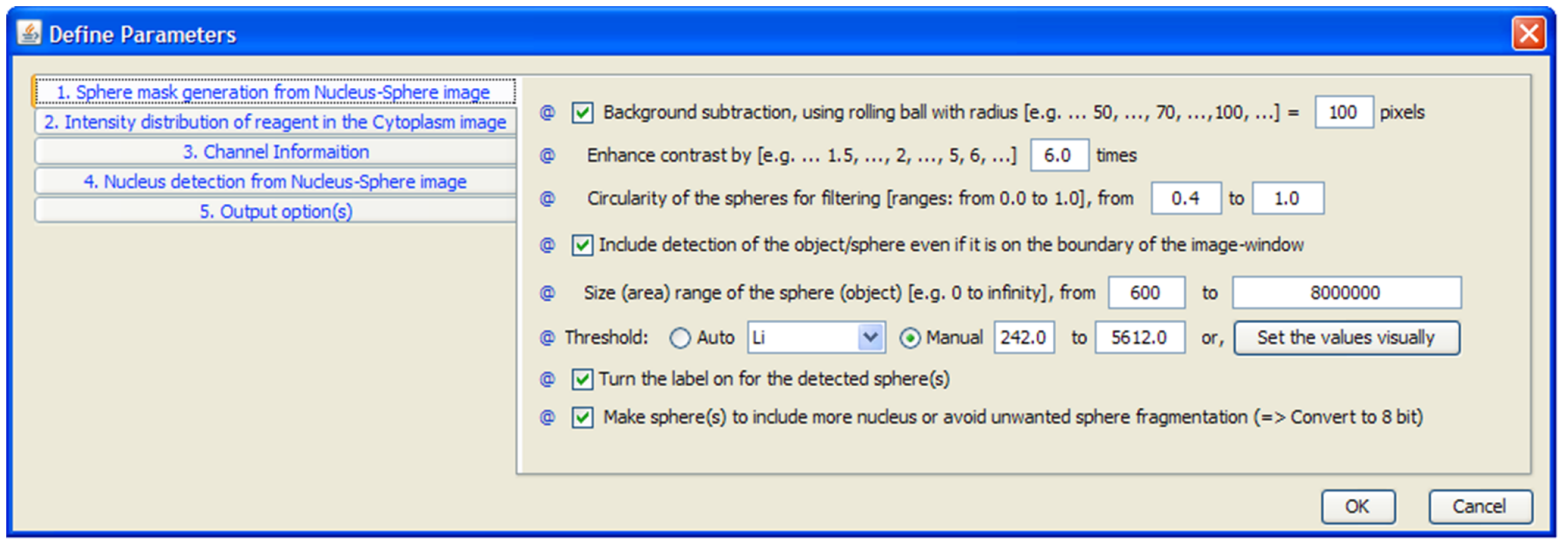
Tabbed-pane for defining parameters. One of the five tabbed sections related to the ‘mask-generation’ is visible.

In addition, an independent FLEX to TIFF convertor was developed to provide images in a more generic format. Many parameters in the *GenericDialog* of ImageJ needed to be accommodated to achieve this. Unfortunately, *GenericDialog* was limited in handling more than a few parameters, thus it was necessary to further combine ImageJ and NetBean (version 6.8) [33] to develop a customised Tabbed-Paned Dialog (Figure 1) for PCaAnalyser. This Tabbed-Paned Dialog is readily and efficiently accommodating almost 30 parameters of different types. The heart of the software is *ParticleAnalyzer* from ImageJ [26]–[28]; however we have extended it further to be used in *batch-mode* to complement the *single-mode* option. ImageJ has been updated accordingly and thus to use our PCaAnalyser as a minimum, ImageJ version 1.44d is required.

Overall, the algorithms of PCaAnalyser can be divided into the following sequences: *i*) overall spheroid detection and mask generation, *ii*) nucleus and membership detection, *iii*) detection and cytoplasm read and *iv*) reporting.

### 2.1 Spheroid Detection and Mask Generation

**Figure 2:**
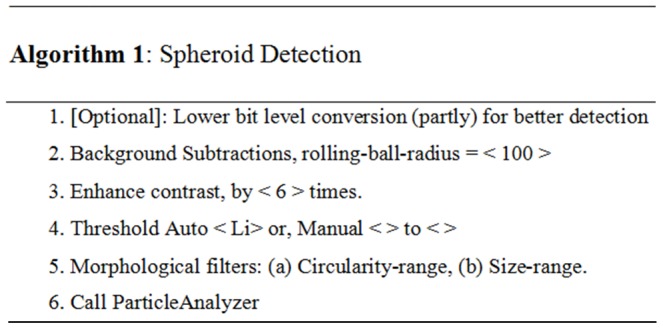
Pseudo code of Algorithm 1. Major steps of the spheroid detection algorithm. Variables with sample values are placed within the angle brackets (i.e. < … >).

**Figure 3:**
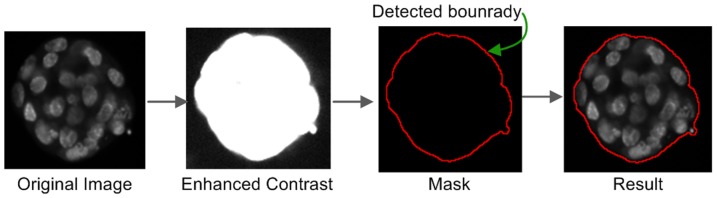
Spheroid detection. The major steps involved in spheroid detection are illustrated.

Ch-1 has the image of the nuclei, grouped per spheroid. Ch-1 to segment is processed to detect the complete spheroid area and boundaries, enabling the formation of the boundary-mask using Algorithm 1 (Figure 2) and the corresponding major steps are shown in Figure 3. The boundary-mask is used for processing images of Ch-2 for: (a) noise removal and (b) to read intensities of cytoplasm and membrane areas, ranging from zero to high values. Ch-2 has very uneven intensities, including values as low as zero for the membrane and cytoplasm area of the spheroid, and also includes many higher intensity and lower levels of SNR. Therefore, Ch-2 cannot be used for boundary detection of the spheroid reliably.

While processing Ch-1, difficulties associated with signals resulting from uneven illumination are experienced. Using the background subtraction with an appropriate radius of the rolling-ball-algorithm, we were able to eliminate this image related artefact. Assuming, the height as a 3rd dimension on a 2D surface of a background image, provides the pixel-intensity proportionally of that image. With the purpose of having a smooth background, the rolling-ball algorithm can assume a ball of chosen radius is rolled over the 2D surface and the hull of volume reached by the ball is the expected smoothened background. In order to accomplish this, first the spheroid-boundary was detected by enhancing the contrast considerably (6 times) to separate the low signals from the background. In the next step, two possible ways were provided for the user to proceed: (a) auto or (b) manual to identify the appropriate contour based on the depth of the original signal-gradient of 3D objects and other morphological parameters, such as circularity and size (area). Options to convert the images into lower bit levels were also provided, which helps to separate the unwanted fragment in the image resulting from uneven illumination caused by the experimental setup.

With the pre-processed and provided parameters, the ParticleAnalyzer was deployed to detect the spheroids – the algorithm was applied to approximately 1000 images and the resultant detection was performed with more than 90% accuracy, when compared to manual microscopy analysis and simple object recognition programmes. Per image, there were generally 10 spheroids on average.

### 2.2 Nucleus and Membership Detection

**Figure 4:**
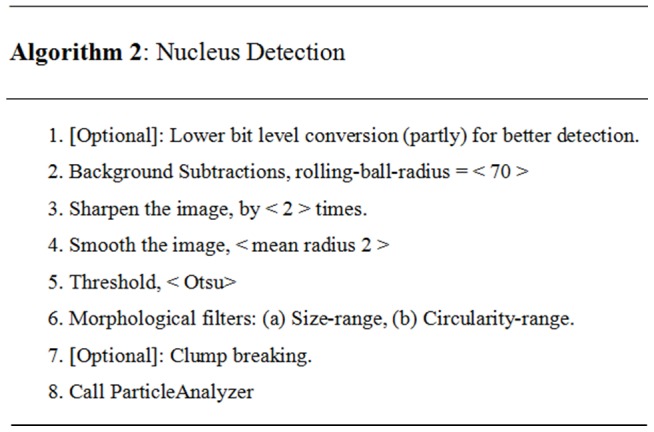
Pseudo code of Algorithm 2. The major steps required for the nucleus detection algorithm. Variables with sample values are placed within the angle brackets (i.e. < …>).

The signal from Ch-1 was used for nucleus detection; however the corresponding image had uneven illumination which impacted on the efficiency of the analysis programme (see ‘Original Image’ in Figure 3). Thus, it was necessary to build and incorporate at least 10 additional parameters to enable accurate and reliable nucleus-detection. The final nucleus detection algorithm (Algorithm 2) developed has been outlined in detail in Figure 4.

**Figure 5:**
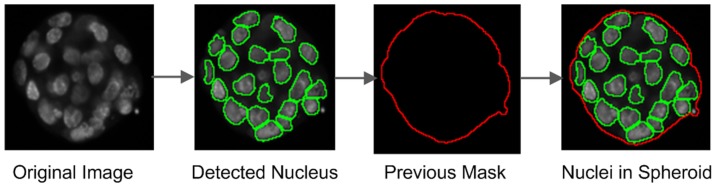
Nucleus-detection. The critical steps involved in nucleus-detection, in addition to performing the task of superimposing previously generated corresponding spheroid boundaries.

The nucleus-image, available in Ch-1 has also been used for spheroid detection. As shown in Algorithm 2, in the first instance, background subtraction was used to decrease uneven illumination, and the image resolution was then sharpened (step 3). Within a given image, not all nuclei were found to have the same height along the z-axis, resulting in some of them being out of focus as they resided in an alternative focal plane within the 3D spheroid. Applying the module ‘enhancement of sharpness’ (ImageJ function), we were able to reduce the number of pixels and thus improve the detection of the given signal. We also applied the ‘smooth operation’ (ImageJ function) module to avoid non-smooth or zigzag type boundary-detection of the nucleus. A suitable threshold-algorithm (step 5) was then applied for segmentation and detection of the nucleus. In addition, the morphological filter was applied to filter out unwanted noise. The steps of this analysis are shown in Figure 5.

**Figure 6:**
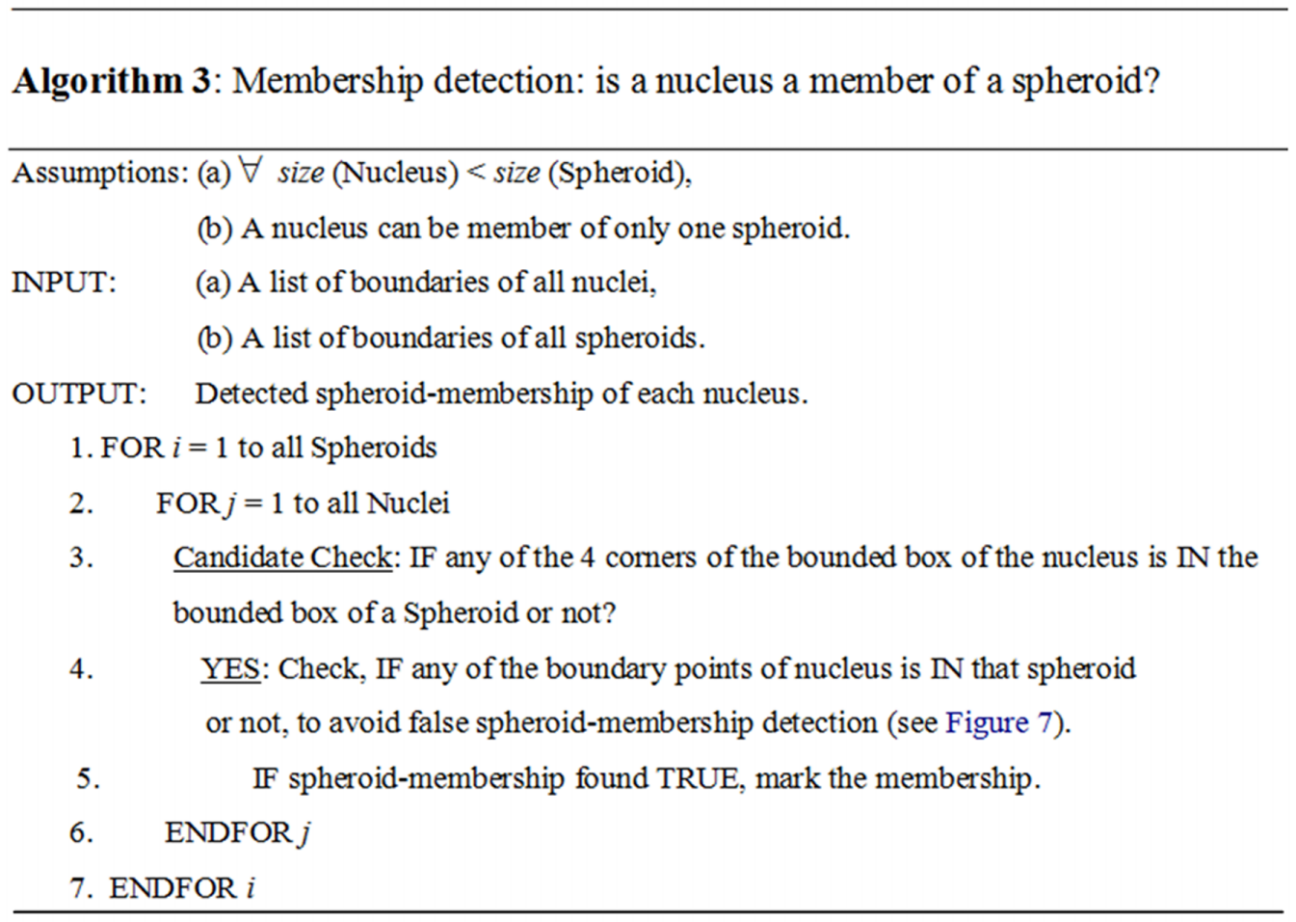
Pseudo code of Algorithm 3. The major steps in detecting spheroid-membership of a nucleus are shown.

**Figure 7:**
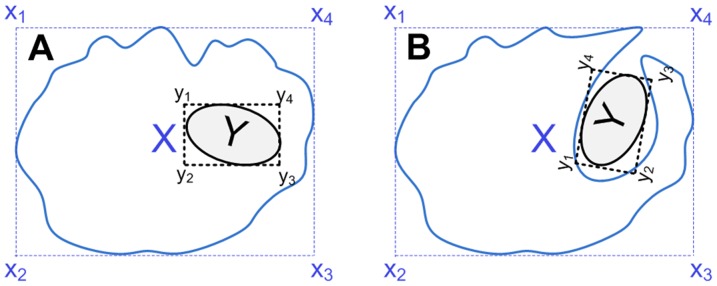
Candidate-checking. To perform the candidate-check in Algorithm 3 to primarily detect whether object Y is possibly inside object X or not, using the bounder-box approach. In the case of (A), object Y is inside object X, therefore, at least one corner of the bounder-box of Y must be inside of the bounder-box of X. However, even if any of the corners of the bounder-box of Y is inside X's, Y may not actually be inside of X, such as, case (B).

In addition to the major steps in detecting spheroid-membership of a nucleus qualitatively (Figure 5), we have simultaneously quantitatively detected the membership. For this, we developed and deployed Algorithm 3 (Figure 6). To perform the candidate-check in Algorithm 3, we employed the bounder-box approach to detect whether object Y is possibly inside object X or not (Figure 7).

### 2.3 Detection and Measurement of Intensities of Membrane and Cytoplasm Areas

The information available through Ch-2 is expected to have various intensity levels (signals) around the membrane and cytoplasmic area of the spheroid. Relevant areas were segmented by generating the boundary-mask of the spheroid in the previous steps (section 2.1). This enabled us to reliably read the lower intensity of the non-background area and to avoid the noisy areas.

**Figure 8:**
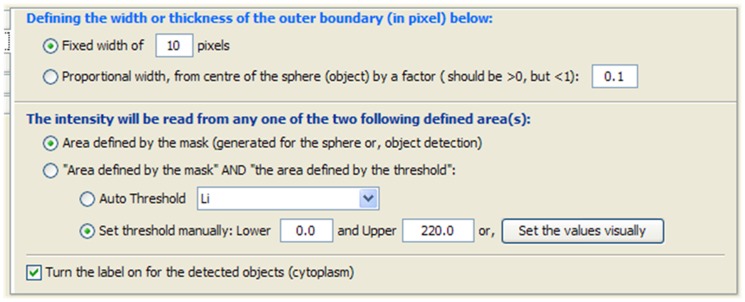
Involved parameter. Measuring intensities and classification of the intensity distribution.

**Figure 9:**
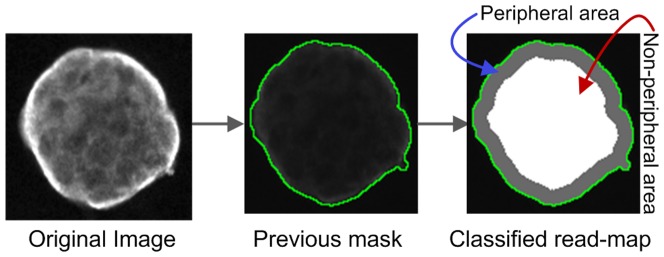
Read-map generation. Major steps involved in generating the classified read-map.

An objective was to analyse cells based not only on the average intensities but also on the distribution of given proteins. Ascertaining whether the expression of proteins reside primarily at the cell-cell junctions, or in the cytoplasm, will help confirm both basal expression levels in cancerous and non-cancerous cells, and to what extent certain treatments have on protein expression. The algorithm involved in measuring intensities and classifying intensity distribution provides 4 possible major combinations (Figure 8) enabling a degree of freedom to study various patterns of intensity distribution, especially important for classifying peripheral and non-peripheral area. We define the segregation of the areas in an automated and reproducible fashion in four possible ways. They are described as:

**i) Define fixed width from boundary and area defined by the boundary-mask.** This combination will read the whole area within the mask and will classify the measured area into two distinct areas: ‘peripheral-area’ of width *x* (variable) pixels inside from the boundary and the remaining non-peripheral area (Figure 9).

**Figure 10:**
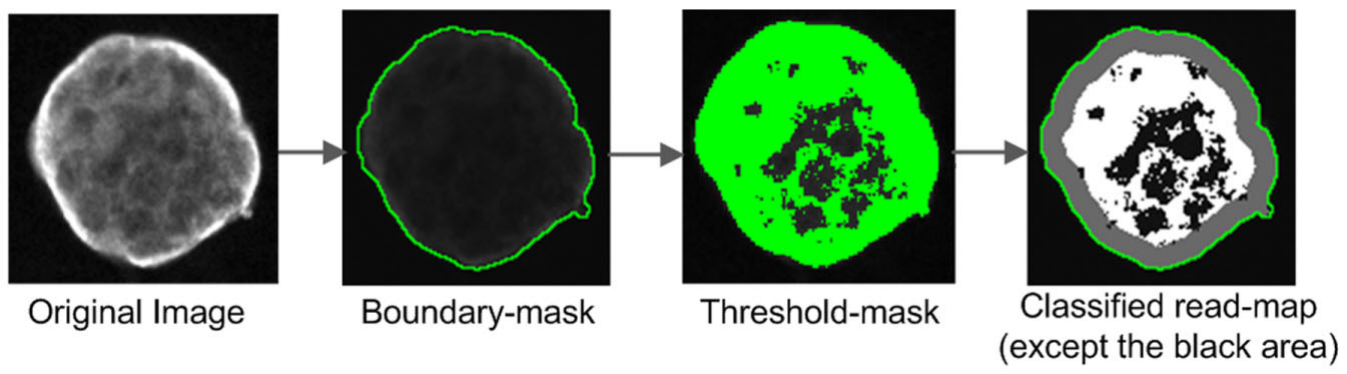
Advanced read-map generation. The major steps involved in generating the classified read-map via boundary-mask and threshold-mask. Images depict a DU145 spheroid grown in a 3D matrix following immuno-labelling for the α6 integrin subunit. Panels in this figure refer to the intensity of the antibody and distribution of the α6 integrin subunit. Labelling was present primarily in the peripheral region of the spheroid structure.

**Figure 11:**
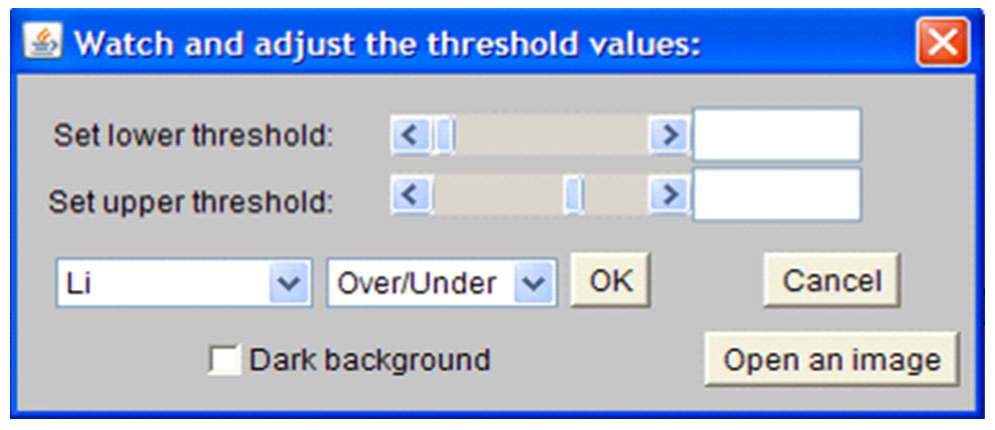
Dialog to set the threshold values visually.

**ii) Define fixed width from boundary and the common area of the mask and the above threshold.** These combinations are similar to the aforementioned option, which is *number*-(*i*), except instead of reading the whole area within the mask, it will take into account those intensities which are above the assigned threshold-value. The major steps are shown in Figure 10. The threshold can be assigned automatically as well as manually using the dialog shown in Figure 11. It is also possible to check the effect visually. A similar dialog is available in ImageJ, however the ImageJ version of the dialog is limited in passing selected threshold-values to the customised plug-ins of PCaAnalyser. Thus, we developed a similar but extended dialog (Figure 11) for PCaAnalyser.

**Figure 12:**
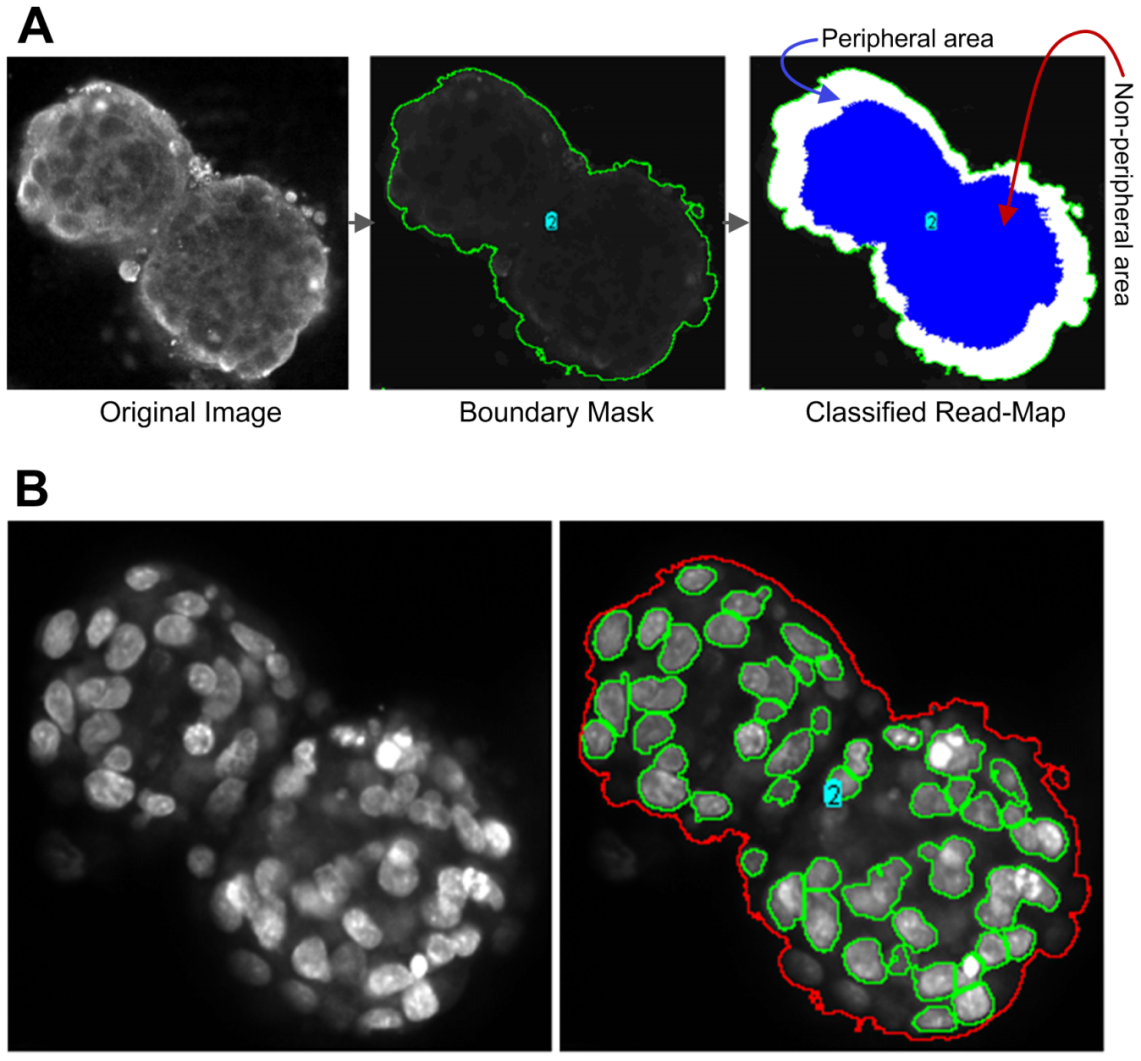
Peripheral versus non-peripheral and clump-breaking defined functions. A) Steps involved in processing read-map classifications with options set for proportional width and boundary-mask. Images depict a DU145 spheroid in a 3D matrix following immuno-labelling for β1 integrin subunit. Panels in this figure refer to the intensity of the antibody and distribution of the β1 integrin subunit. The distribution of β1 remained primarily around the outer membrane/peripheral region of the spheroid. B) Original image of DU145 spheroid B') Application of PCaAnalyser utilising the clump-breaking functions, after magnifying the signal the software found non-zero signals in between the two spheroids like masses and detected it as a single spheroid.

**iii) Proportional width from centre of the spheroid (object) by a factor y (where,** 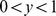 **) and area defined by the boundary-mask.** Unlike the fixed width, this option first determines the centre of the object and then applies proportional width to classify a pixel based on whether it belongs to peripheral or to the non- peripheral area. The process is shown in Figure 12A.

The peripheral versus non-peripheral function (Figure. 12A) was particularly useful for investigating the Chemokine receptor, CXCR4, expression and distribution in response to ligand treatment. Our objective was to evaluate whether there were differences in expression in both the absence and presence of its ligand, SDF-1α. In the absence of SDF1α, the CXCR4 protein was only expressed on the peripheral regions of the spheroids. After treatment, however, the CXCR4 expression changes and migrates further into the middle of the spheroid and was found within the non-peripheral regions. Therefore, this analysis allows validation as to whether or not a protein is functional in the 3D cell culture model system, or not.

**iv) Proportional width from centre of the spheroid (object) by a factor y (where,** 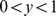 **) and area defined by the boundary-mask and the threshold-mask.** This is the same as the immediate previous combination (*number*-(*iii*)), with the exception that the read-map excludes those pixels that are below the (upper) value of the assigned threshold-mask.

Visually, the image depicted in Figure 12A could possibly be viewed as two separate spheroids in close proximity to one another. However, it is known that over time in culture, spheroids can merge and fuse together to form larger masses [3]. It was therefore imperative to formulate a process that could verify a single vs fused object. We accomplished this via a feature called “false clump-breaking” candidate. This feature helps the PCaAnalyser software to determine whether spheroids are truly connected or not. The false clump-breaking is difficult to detect visually, however the PCaAnalyser solves this problem by amplifying the signal to more clearly define the situation where low signal exists (i.e., false clump-breaking candidate) versus no signal exists (i.e., true clump-breaking candidate). The principle is that the amplification of ‘no signal’ will remain zero. Figure 12B represents such a situation where the software identifies it as a false clump-breaking candidate whereas visually it appears to be a true candidate.

### 3. Analysis of PCa DU145 and morphometrically diverse PC3 cells

Using immuno-cytochemistry procedures, we analysed the expression patterns of integrin α6 and β1 subunits on DU145 cells. Furthermore, we analysed the intensity of β1 subunits on PC3 cell-lines in the presence and absence of DHT in 3D cultures. Twenty-four wells of a 96-well-plate were analysed by evaluating two channels with 20 images captured per channel. Thus, over 1000 distinct images were processed for each analysis. Each of these images contained ∼10 detected spheroids and approximately 10 to 40 nuclei detected per spheroid.

In addition, for proof of principle, we undertook analysis of a metastatic BCa cell line MDA-MB-231 taken with a ×10 objective. Here we analysed the expression of the integrin β1 subunit in response to treatment with Doxorubicin, a well-known therapeutic used in a range of cancer treatments.

While processing the images using PCaAnalyser, a comprehensive array of measurement properties and object details were automatically exported to a database where analytic reports could then be generated.

The performance of the software was robust, as: (a) it performed well in a very noisy environment and (b) selection of the cut-off value defined to enable inclusion of nuclei was simplified.

**Figure 13:**
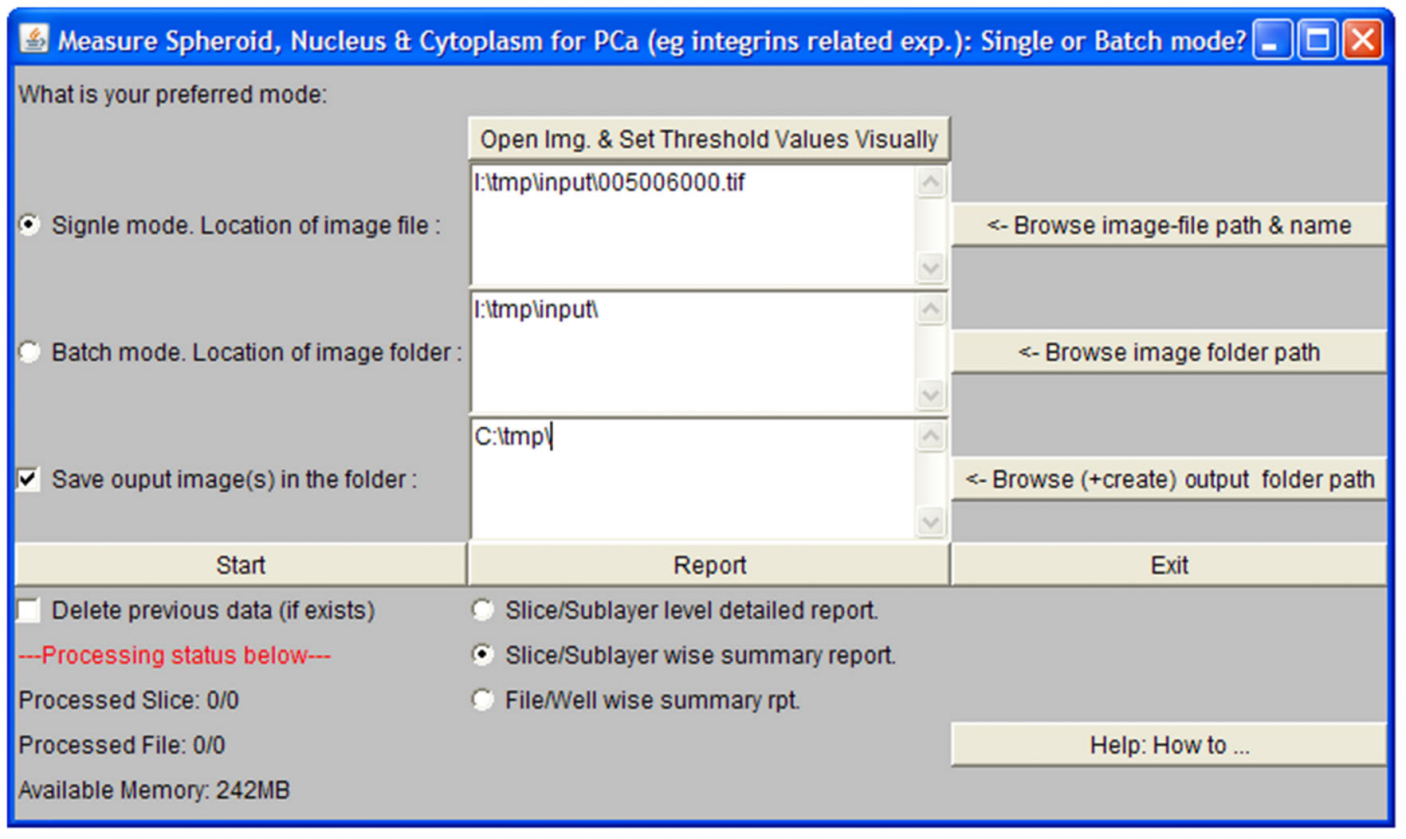
The main interface of *PCaAnalyser*. *PCaAnalyser* is offering both *single mode* and *batch mode* of operations and offering at least 3 different levels of reporting.

The software can be operated in single mode for a single file representing a single well, or alternatively in batch mode through the simple interface outlined in figure 13 (Figure 13). Each image file can be of a single image or a stack of images, where each single image represents a single sub-layer within a well.

All raw data is stored in an Access database. Generated reports are provided in *comma-separated-value* (CSV) as well as in jasper file formats. The reports are also arranged in tabular format with the row-column being the same as the experimental-plates. In addition, files are named according to their well location.

### 3. 1 Software Processing

**Figure 14:**
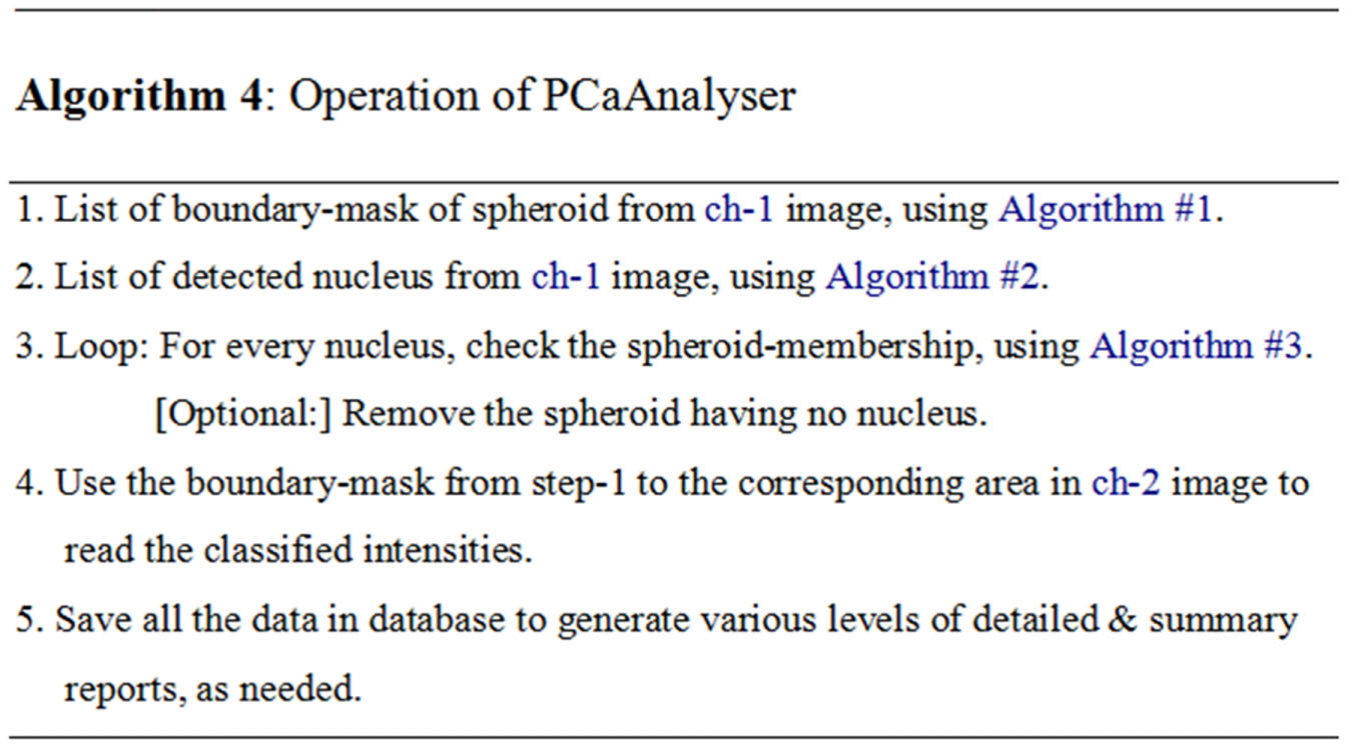
Pseudo code of Algorithm 4. Central functions of PCaAnalyser, showing the major steps: calling algorithms 1–3 and integrating the database.

**Figure 15:**
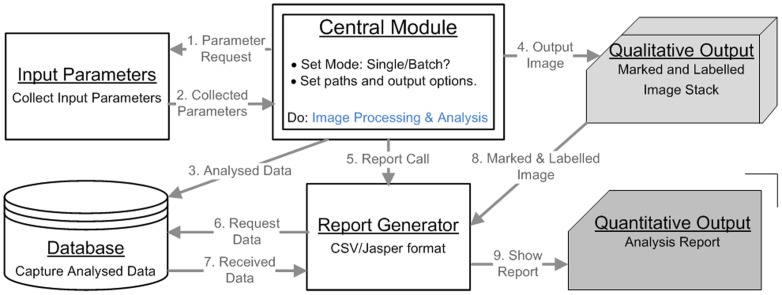
Operational overview of PCaAnalyser software. Paradigm and flow-diagram of PCaAnalyser, depicting the integration and operation sequences of inputs, central-operations, data-processing, output-generation and output-formats.

The implicit operations of the software are summarised in Algorithm 4 and shown in Figure 14. The explicit operations of the software, along with software-architecture, are depicted in Figure 15.

The software, PCaAnalyser, is a plug-in for ImageJ and has been developed in java using Neatbean (Ver. 6.8) and Microsoft Visual-J# 2005 editors. The access database has been used for capturing analysed data, the architectural outline for this is given in Figure 15.

### 3.2 Software Output and Quality Assessment

The software can generate two different outputs:

- *Quantitative*: extracted features from the image analysis are captured in a database. Various levels of report views are available based on this database.
- *Qualitative*: output images with various labels, colours and read-maps are inserted adjacent to input image forming a stack, which can conveniently allow immediate comparisons of input versus output images.

**Figure 16:**
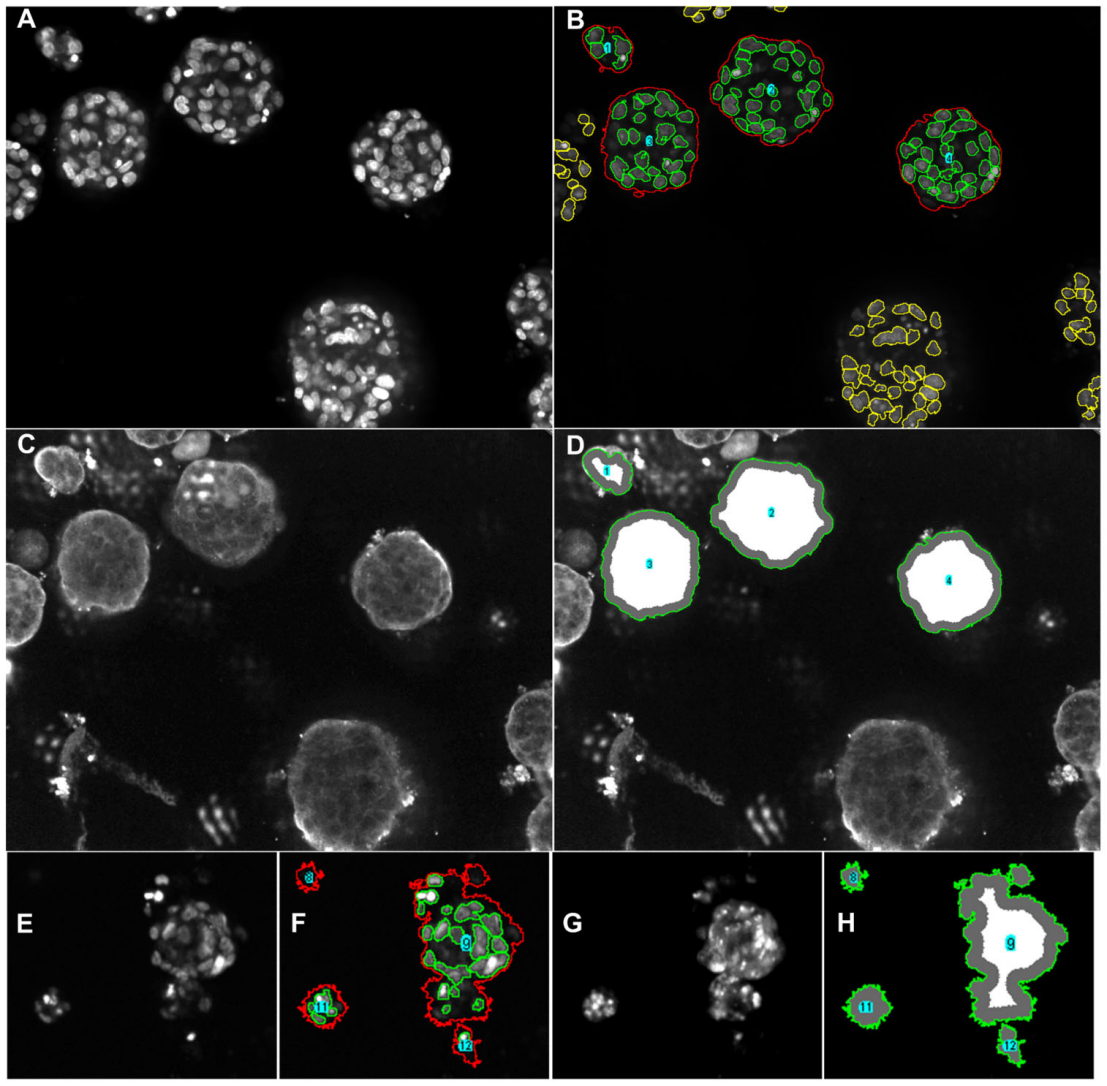
Overall outcome from the application of PCaAnalyser software. Panels show DU145 and MDA-MB-231 spheroids grown in a 3D matrix following immuno-staining for dapi (nucleus; A, E), Beta 1 integrin (C) and doxorubicin (G). (A) Original image of DU145 spheroid and nucleus. (B) Detected spheroid and detected nucleus. The spheroids interacting window-boundary has been optionally chosen to be excluded, and in such a case the corresponding detected nucleus is shown in a different colour (yellow). (C) Original cytoplasm and membrane area image, having noise of higher intensities as well as quantities. (D) Classified read-areas for studying intensities are highlighted and the distinct spheroids are optionally labelled. The generated mask from (B) helps avoid noise effectively and a fixed width from peripheral-boundary has been selected in this case to generate the classification. (E–H) MDA-MB-231 spheroids treated with doxorubicin and imaged with a ×10 objective. (E) Original image of MDA-MB-231 spheroid and nucleus. (F) Detected spheroid and detected nucleus (G) Original image of doxorubicin staining. (H) Classified read-areas for studying intensities.

**Figure 17:**
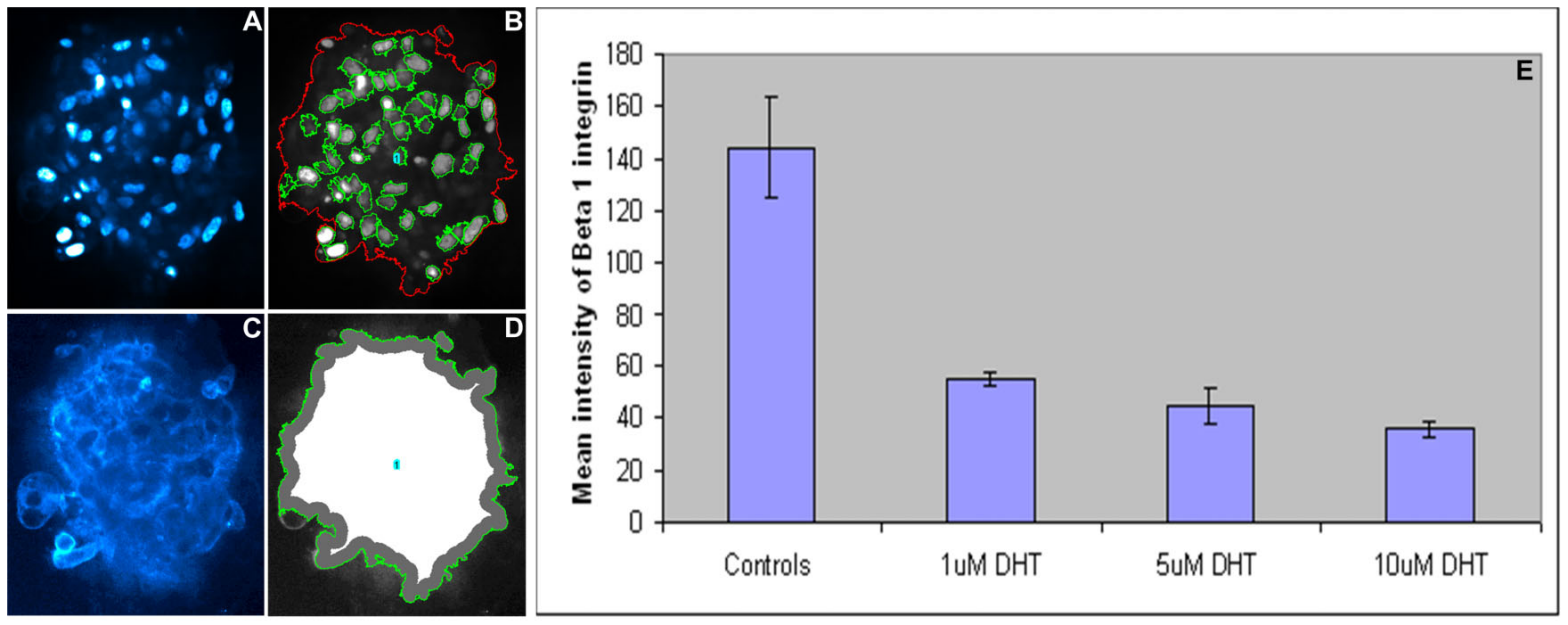
Nucleus and beta 1 integrin detection in morphometrically diverse PC3 cells. Panels show PC3 spheroids grown in a 3D matrix following immuno-staining for dapi (nucleus; A) and Beta 1 integrin (C). (A) Original image of PC3 spheroid and nucleus. (B) Detected spheroid and detected nucleus. (C) Original cytoplasm and membrane area image. (D) Classified read-areas for studying intensities. (E) Quantitation of Beta 1 intensities in PC3 cells treated with DHT.

Irrespective of resolution acquisition (×10 or ×20 objective), once processed, the quality of the software output is evident from the sample image in Figure 16. We next investigated whether PCaAnalyser could adequately evaluate the intensity of β1 integrin expression using morphometrically diverse metastatic PC3 cells in the absence or presence of DHT (Figure 17). Similar to the results obtained for DU145 cells, when processed with PCaAnalyser, both the nucleus (Figure 17A–B) and β1 expression (Figure 17C–D) could be detected and quantified in a reliable and reproducible manner. Utilising a sublayer wise report output, we could successful quantify the effects of DHT treatment on β1 integrin expression in PC3 cells. Treatment with 1–10 µM of DHT resulted in a significant dose dependent decrease in the general intensity of β1 integrin (Figure 17E). These results suggest that non-genomic AR can mediate β1 integrin expression in this metastatic PCa cell line.

### 3.3 Output Reporting

**Figure 18:**
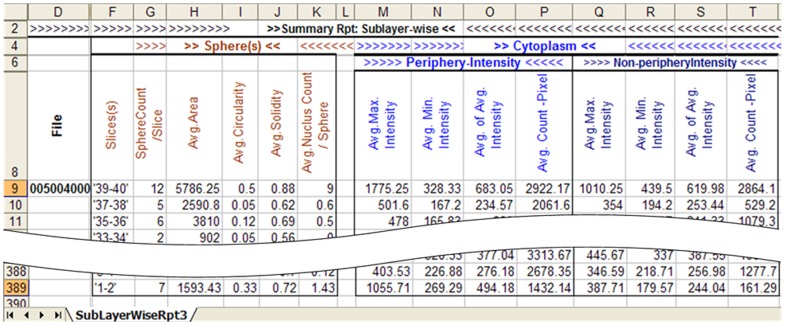
Sample report. Sub-layer-wise summary ∼.csv report.

For output reporting, the CSV (Comma-separated values) format (see Figure 18), as well as jasper report, have been used. Java-scripting and SQL-scripting were also utilised in report generation. The reasons for using the CSV report format are: (*i*) CSV can be conveniently used to interface between modules, i.e. convenient for future extensions, (*ii*) Jasper-report could generate CSV, but it would need 2 passes and often the column-alignments were incorrect when converted to CSV format from jasper-report, (*iii*) the CSV file would allow integration with our in-house built general purpose data-analysis software and finally (*iv*) as an excel application, CSV allows users to conveniently apply statistical functions as required.

On the other hand, incorporation of jasper-report enabled immediate amalgamation of qualitative (i.e., image) as well as quantitative data, for final reports. Both the report formats provide various levels of analysis and include: (*i*) detailed analysis reporting of any single image (from a single sub-layer within a well) and (*ii*) the corresponding summary report of (*i*), (*iii*) summary on a file, containing all the sub-layers within a well and (*iv*) micro-titre plate formatted summary report based on a single property of the experimental quest.

The PCaAnalyser tool is freely available and includes a user's guide, generated codes and sample images [43].

## Discussions and Conclusions

We present the first software that is capable of analysing 3D cell spheroid data in an automated and reliable fashion, and is readily accessible. The software development has been described for metastatic PCa DU145 (see Figure 16) and morphometrically diverse PC3 cells (see Figure 17). The expression patterns concerning protein constituents known to be involved in regulating the progression of PCa have been analysed, and include the integrin α6-β1 sub-unit and chemokine receptor, CXCR4, expression. We have also shown that this software can be extended and applied to other 3D cell model systems, as evidenced by the successful analysis of the BCa cell-line MDA-MB-231 (see Figure 16).

### 

#### 1. Expression patterns of Integrins and CXCR4 in PCa DU145 and PC3 cells

Consistent with a highly invasive phenotype, we have shown that in 3D DU145 cells in 3D express functional CXCR4 and are similar to metastatic prostate cell biopsies, with high levels linked to more aggressive phenotypes and the extent of metastasis [34], [35]. CXCR4 expression was found primarily on the outer membrane of DU145 spheroids, while treatment with SDF-1α resulted in a re-distribution of CXCR4 to the centre of the spheroid, consistent with active ligand-induced recycling.

Similarly, we have shown both PC3 and DU145 cell-lines expressed membrane bound integrin β1 which is similar to metastatic prostate cell biopsies, with high levels linked to more aggressive phenotypes [34], [35]. Recent studies have demonstrated that the β1 integrin subunit controls the growth and invasion of prostate tumour cells in 3D culture conditions [36], [37] and knockout strategies in transgenic mouse tumour models have shown that integrins control primary tumour growth and dictate the site of metastatic spread [38]. Interestingly, these influences are largely masked by growth of tumour cells in the standard environment of 2D cell culture due to the lack of cell-cell and cell-ECM complexity [36].

Previously, we have demonstrated that PC3 cells cultured in 3D re-express non-transcriptionally active AR [4]. Here we present data that suggests that non-genomic AR can mediate Beta β1 integrin expression in this metastatic cell line. These results are consistent with the findings of others where non-genomic effects of androgens influence the activation of kinase cascades and cytoskeletal rearrangement [22]–[24]. Functionally these results correspond with the pathophysiological progression of PCa. At onset, AR is upregulated in the prostate which is known to alter a range of protein constituents including integrins [39]. Down regulation of β1 integrin have been associated with increased dissemination of tumour cells from the primary epithelium [39]. These results have far reaching implications regarding late stage therapeutics and further studies are now needed to evaluate additional non-genomic effects of AR regulation in PCa progression.

The ImageJ based PCaAnalyser provides many degrees of freedom that has enabled us to address the challenges set out in section 2.3. As can be seen from the quality of the input image (Figure 16A) and corresponding output image (Figure 16B), the detection of the spheroid boundary, as well as the boundary of the nucleus, was successfully analysed from the same channel. The corresponding intensity-read was successfully performed within a noisy environment, as can be observed from input (Figure 16C) and output (Figure 16D). The same is also true for Figure 16E–H and for Figure 17A–D applied to the BCa MDA-MB-231 and PCa PC3 cell-lines, respectively.

The flexibility to include, or exclude, the nucleus that is visually blurred can be manipulated by adjusting the parameters of algorithm 2 (Figure 4). We basically sharpened the image to include the nuclei that are not in the same depth. This could also have been done using the ‘shrink and grow’ based approach to make the blurred images sharper. Further, those nuclei that are at the side of the image of the 3D spheroid have an angulated view, compared to the nuclei which are relatively central within the image. This effect causes uneven illumination within the same spheroid. As this is a physical property attributed to the 3D environment of the spheroid itself, even if the nuclei are co-planar, the angulated nucleus emits light less perpendicularly towards the imaging CCD camera, and thus the angulated nuclei are observed as being darker. As a future step, the ‘affine region detection’ based approach [40] can be considered to further improve the processing of such cases.

### 2. Efficiency of Algorithm 3

The computation of the spheroid-membership-check of the nucleus are computationally time intensive. To make the membership checking faster using Algorithm 3 (Figure 6), we initially applied bounded-box based candidate checking. Here, we compute the time complexity to measure how much we have improved the speed of analysis. Assume in a single image, we have, *m* = numbers of spheroid on an average and *n* = numbers of nucleus on an average, *x* = is the number of pixels on an average forming the boundary of a nucleus. Therefore, using algorithm 3 without bounded-box checking steps, we can estimate the average operations as:
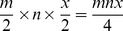
(1)where, to get a membership it is assumed that we have to traverse half of the spheroid list (i.e., 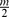) on an average and half of the boundary pixel (i.e., 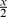) of the nucleus on average. Obviously, the time complexity would be at least 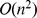. Now, using the algorithm #3, as it is, with the bounded-box checking option on, the rate of *true* membership found from bounded-box is assumed to be *y*%. The involved operations can be estimated as:
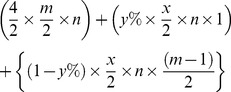
(2)At the beginning of Equation (2), we are considering 2 corner-points checking on an average out of 4 corner-points of a bounded-box. For *y*%, practically we found that practically it tends to 100% and thus the 3^rd^ part of Equation (2) is eliminated. Therefore, Equation (2) can be expressed simply as (3):
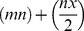
(3)Thus, the speed-up due to having the candidate-check can be estimated using Equation (4), formed by Equation (1) over Equation (3):
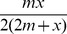
(4)From our experiments, using average typical values of *m* = 10 and *x* = 100, using Equation (4), the algorithm was found to be 4.16 times faster due to the candidate-check step.

### 3. Adaptation to the Parallel Execution

ImageJ based software was found to be reasonably fast (16 times faster on average) when compared to the time taken for the same number of operations performed by the software associated with the Opera, a high throughput confocal image which was used for image acquisition. Based on the anticipated high volume of screening to be undertaken, the software architecture (Figure 15) was built to perform in a distributed and parallel manner.

The paradigm of PCaAnalyser supports multiple instances running on the same computer sourcing data from a single database. For example, images are stored in a separate folder based on the different source plates, and different instances of PCaAnalyser can be used to analyse the data from individual folders simultaneously. To apply the idea, if previous data are to be deleted, only the first instance will need to turn on the *delete* option (i.e. the option, ‘Delete previous data (if exists)’ in Figure 13). The separate instances of the PCaAnalyser can be executed to process separate folders containing datasets. Alternatively, an outer loop can be added within the code to process more than one folder as required, which would be a relatively simple modification.

Within a network environment having multiple computers, the processing capacity can be easily scaled-up by having the instances running in parallel on every computer. However, the database can either be uniquely pointed to a single place or otherwise different databases can be merged simply by copying and pasting data to integrate within one master database.

The option for further enhancing the processing capacities can be made [41] by involving GPU [42] which are now-a-days more commonly available with a powerful graphics card, such as AMD Radeon or NVIDIA GeForce.

### 4. Classification Functions for Intensity Read

Four possible major combinations for the classified reading of the intensity of the spheroid have been provided in section 2.4.3. The peripheral regions from non-peripheral regions were independently defined to separate events (proteins) that may be localised to either/or both of these regions. The ability to distinguish between the two locations is important as this allows us to also measure functional translocation events.

Also, for the relatively elongated or ellipsoid cases, we defined a sophisticated function to segregate more areas at the two elongated ends to study whether increasing integrin accumulation in this phase was associated with accelerated PCa progression or not.

### 5. Conclusions

Finally, we report that PCaAnalyser is an effective, and extendable analytical tool for high throughput analysis of images acquired from cells grown in a 3D matrix. We have shown that the software can reproducibly analyse immuno-staining of different markers known to be involved in cancer progression including CXCR4, α6 and β1 integrin subunits. Moreover, we have reported the effects of such protein expression in response to both ligand and drug treatment and at acquisitions of varying resolution acquisition (×10 and ×20 objectives) and clarity. Specifically, PCaAnalyser has been demonstrated to confirm the impact of treatments and their effects on the distribution and intensity of key biomarkers and proteins of interest.
